# A Manual of General Pathology

**Published:** 1876-05

**Authors:** 


### Bibliographical.

---

A Manual of General Pathology. By Ernst Wagner, M.D., Professor of General Pathology and Pathological Anatomy University of Leipzig. Translated from the sixth German edition, by John Van Duyn, A.M., M.D., Professor of Anatomy and Histology in Medical School, Syracuse, N. Y., and E. C. Seguin, M.D., Clinical Professor of Diseases of Mind and Nervous System, College of Physicians and Surgeons, N. J. New York: William Wood & Co. 1876. Book, pp. 727.

This work is composed of four divisions. Part first treats of general nosology; second, general ætiology; third, general pathological anatomy and physiology; fourth, pathology of blood.

Upon a hasty perusal of this work, we are struck with the able manner in which the different parts are investigated and made practical. It is the duty of the profession to search after truth to the very utmost, and as a guide to pathological anatomy we recommend the above work.

